# Association of Opioid Use Disorder Treatment With Alcohol-Related Acute Events

**DOI:** 10.1001/jamanetworkopen.2021.0061

**Published:** 2021-02-24

**Authors:** Kevin Y. Xu, Ned Presnall, Carrie M. Mintz, Jacob T. Borodovsky, Nisha R. Bhat, Laura J. Bierut, Richard A. Grucza

**Affiliations:** 1 Health and Behavior Research Center, Department of Psychiatry, Washington University School of Medicine in St Louis, St Louis, Missouri; 2 Now with Center for Technology and Behavioral Health, Department of Biomedical Data Science, Geisel School of Medicine at Dartmouth, Lebanon, New Hampshire; 3 Alvin J Siteman Cancer Center, Barnes Jewish Hospital and Washington University School of Medicine in St Louis, St Louis, Missouri; 4 Department of Family and Community Medicine, St Louis University, St Louis, Missouri; 5 Department of Health and Clinical Outcomes Research, St Louis University, St Louis, Missouri

## Abstract

**Importance:**

Persons with opioid use disorder (OUD) and co-occurring alcohol use disorder (AUD) are understudied and undertreated. It is unknown whether the use of medications to treat OUD is associated with reduced risk of alcohol-related morbidity.

**Objective:**

To determine whether the use of OUD medications is associated with decreased risk for alcohol-related falls, injuries, and poisonings in persons with OUD with and without co-occurring AUD.

**Design, Setting, and Participants:**

This recurrent-event, case-control, cohort study used prescription claims from IBM MarketScan insurance databases from January 1, 2006, to December 31, 2016. The sample included persons aged 12 to 64 years in the US with an OUD diagnosis and taking OUD medication who had at least 1 alcohol-related admission. The unit of observation was person-day. Data analysis was performed from June 26 through September 28, 2020.

**Exposures:**

Days of active OUD medication prescriptions, with either agonist (ie, buprenorphine or methadone) or antagonist (ie, oral or extended-release naltrexone) treatments compared with days without OUD prescriptions.

**Main Outcomes and Measures:**

The primary outcome was admission for any acute alcohol-related event defined by *International Classification of Diseases, Ninth Revision* and *International Statistical Classification of Diseases and Related Health Problems, Tenth Revision* codes. Conditional logistic regression was used to compare OUD medication use between days with and without an alcohol-related event. Stratified analyses were conducted between patients with OUD with and without a recent AUD diagnostic code.

**Results:**

There were 8 424 214 person-days of observation time among 13 335 participants who received OUD medications and experienced an alcohol-related admission (mean [SD] age, 33.1 [13.1] years; 5884 female participants [44.1%]). Agonist treatments (buprenorphine and methadone) were associated with reductions in the odds of any alcohol-related acute event compared with nontreatment days, with a 43% reduction for buprenorphine (odds ratio [OR], 0.57; 95% CI, 0.52-0.61) and a 66% reduction for methadone (OR, 0.34; 95% CI, 0.26-0.45). The antagonist treatment naltrexone was associated with reductions in alcohol-related acute events compared with nonmedication days, with a 37% reduction for extended-release naltrexone (OR, 0.63; 95% CI, 0.52-0.76) and a 16% reduction for oral naltrexone (OR, 0.84; 95% CI, 0.76-0.93). Naltrexone use was more prevalent among patients with OUD with recent AUD claims than their peers without AUD claims.

**Conclusions and Relevance:**

These findings suggest that OUD medication is associated with fewer admissions for alcohol-related acute events in patients with OUD with co-occurring AUD.

## Key Points

**Question:**

Are opioid use disorder (OUD) medications associated with alcohol-related poisonings, falls, and injuries in persons with OUD and co-occurring alcohol use?

**Findings:**

This case-control cohort study of 13 335 individuals with OUD from a large, nationally representative data set found that patients prescribed OUD medication had significant reductions in alcohol-related acute events.

**Meaning:**

The use of OUD medication in opioid-dependent patients with co-occurring alcohol use may hold promise in reducing alcohol-related acute events, but more investigation is needed.

## Teaser

This case-control cohort study examines whether the use of medications to treat opioid use disorder is associated with decreased risk for alcohol-related falls or injuries and poisonings in persons with opioid use disorder with and without co-occurring alcohol use disorder.

## Introduction

Over the last decade, an unprecedented epidemic of drug-related poisonings has affected countries spanning North America and Europe. Recent estimates of unintentional poisoning deaths (19.1 deaths per 100 000) have eclipsed those of falls (11.4 deaths per 100 000) and motor vehicle traffic deaths (11.6 deaths per 100 000).^1^ This is evidenced by an all-time high of 70 980 overdose deaths in the US in 2019.^2^ Although the medical community has largely focused on the central role of opioid use disorder (OUD), polysubstance use has been underrecognized, even though most patients with substance use disorder use multiple substances,^3^ and the presence of multiple substance use disorders is associated with worse treatment outcomes.^4,5,6^

Alcohol is the most commonly misused substance, both as a standalone drug and in the context of polysubstance use.^5^ More than 25% of patients with OUD exhibit problematic alcohol use, and, therefore, mitigation of risk for incident or recurrent alcohol use disorder (AUD) presents a salient problem in the treatment of OUD.^5,7,8,9^ As central nervous system depressants,^10^ alcohol and opioids can be lethal in combination, leading to increased overdoses and mortality among patients with OUD,^11,12^ in addition to increased health care utilization.^13,14,15^ Because problematic alcohol and opioid use are traditionally studied as independent conditions, limited evidence-based strategies exist for mitigation of alcohol-related risk among patients with OUD.

Despite the therapeutic challenge of treating alcohol and opioid co-use, there is reason to believe that existing OUD medications may hold promise in treating comorbid AUD, thus potentially reducing alcohol-related risk among patients with AUD. For instance, some treatment options for dual-diagnosis patients overlap, such as naltrexone, which holds dual US Food and Drug Administration (FDA) indications for OUD and AUD treatment and has been found to be used more commonly in patients with both OUD and AUD than patients with OUD but without comorbid AUD (Mintz CM, Presnall NJ, Xu KY, et al, unpublished data, November 2020). However, few studies have directly evaluated the efficacy of OUD medications on alcohol-related outcomes among patients with OUD,^15^ and the limited research base has been marked by inconsistent results. For instance, although an open randomized study^16^ suggested that methadone and buprenorphine suppressed alcohol intake in heroin users, other analyses^17^ have not replicated these effects, with some studies^18^ showing increased problematic use of alcohol after initiation of opioid agonist treatment. Existing studies^16,17,19^ are also characterized by short follow-up periods, modest sample sizes, and self-reported data.

Given the research gaps on alcohol-related outcomes among patients with OUD, our study used the IBM MarketScan Commercial and Multi-State Medicaid databases (2006-2016) to evaluate the association of 3 OUD medication treatments—buprenorphine, methadone, and naltrexone—with emergency and inpatient admissions for acute alcohol-related events, specifically falls, injuries, and poisonings in patients with OUD.

## Methods

### Study Overview

This case-control cohort study used a repeated-event, case-control design using the MarketScan pharmaceutical claims data. We studied whether alcohol-related acute medical events were associated with OUD pharmacologic treatment. This design used each subject as his or her own control by comparing OUD medication use (exposure) at the time of event with exposure during control periods. Thus, the exposure variable was various types of OUD pharmacologic treatments, and the outcome variable was alcohol-related acute events. We also examined whether patients with known recent AUD claims were more likely to receive naltrexone, given its dual FDA indications for AUD and OUD, as opposed to buprenorphine and methadone, and whether the association between OUD medication and alcohol-related events varies by the presence of recent AUD claims.

### Study Setting

The MarketScan databases represent claims from both employer-sponsored and Medicaid insurance holders in the US.^20^ This captures longitudinal information on inpatient and outpatient health care utilization, diagnosis codes based on the *International Classification of Diseases, Ninth Revision (ICD-9)* and *International Statistical Classification of Diseases and Related Health Problems, Tenth Revision (ICD-10)*, and procedure codes. Prescription medication data provided information on medication quantity, dosing, and fill dates. Commercial data were available from January 1, 2006, to December 31, 2016. Medicaid data were available from January 1, 2011, to December 31, 2016. Because all data were deidentified, our study was exempt from human subjects review and the need for informed consent by the institutional review board at Washington University. This study follows the Strengthening the Reporting of Observational Studies in Epidemiology (STROBE) and RECORD-PE reporting guidelines.

### Participants

**Figure 1.**
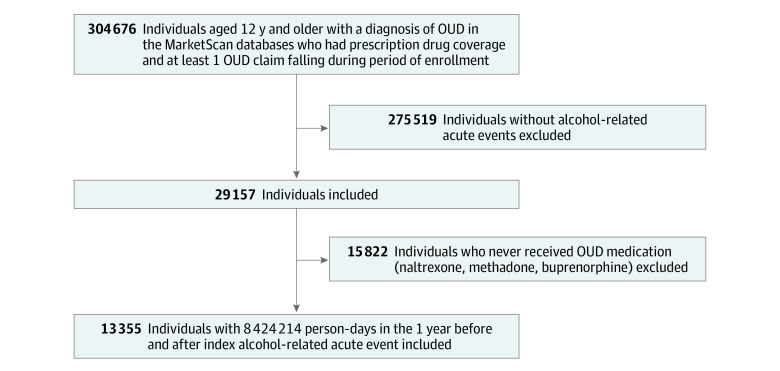
Flowchart for Development of Analytical Sample Chart shows derivation of the study’s final analytical sample during follow-up. After application of inclusion and exclusion criteria, there were 13 335 unique patients with opioid use disorder (OUD), all of whom had received OUD medication during insurance enrollment. The analytical sample was subsequently restricted to observations within 1 year before and 1 year after index alcohol-related acute event to decrease heterogeneity in observation time, contributing a total of 8 424 214 person-days in the study database.

Our analytic sample was derived from a previously described cohort of 304 676 individuals in the US, aged 12 to 64 years, who had prescription drug coverage, a diagnosis of OUD, at least 1 OUD claim during enrollment, and continuous insurance enrollment.^20^ Figure 1 depicts the derivation of our final analytical sample. Because we used a case-control design, we limited analyses to the 29 157 individuals with alcohol-related acute events (ie, 275 519 individuals were ineligible because they did not experience the outcome of interest). The first event for each person subsequent to initiating OUD treatment was defined as the index event. The index event date was used to generate a longitudinal data set at the day level containing all days in which a given individual was observed (ie, covered by insurance), up to 1 year before and after the index event; that is, the final data set contained 1 record per day per person, with the units of observation being person-days. Our decision to choose a 2-year maximum observation window was guided by the mean length of time individuals were observed in our data set, which was approximately 2 years. The selection of a standard, 2-year observation window also helps fulfill the case-control design’s requirement of a stable within-person probability of exposure over a defined period.^21^

Finally, we further limited our analyses to individuals who received OUD medication at least once, thus excluding 15 822 individuals who never received any pharmacotherapy for OUD (buprenorphine, naltrexone, and methadone). Participants were permitted to contribute multiple events as long as they fell within a maximum of 1 year before and after the index event.

### Variables

Our primary outcome was emergency department visit or inpatient hospitalization for any alcohol-related acute event during insurance enrollment. Such events were defined on the basis of established methods and *ICD-9* and *ICD-10 *codes^22^ as either (1) 100% alcohol-attributable acute events (eg, alcohol poisoning) or (2) health care encounters involving an alcohol-related diagnosis (eg, alcoholic liver disease or chronic alcohol dependence) in combination with acute events that are known to be associated with excess alcohol use (eg, falls, injuries, and poisonings).^22^

Our primary exposure variable was OUD medication by days, operationalized as days characterized by the presence or absence of prescription coverage or procedure codes for buprenorphine, methadone, or naltrexone (oral or extended-release). Specific national drug codes (buprenorphine and oral naltrexone) and procedure codes (methadone and extended-release naltrexone) can be found elsewhere.^20^ We assumed that the prescription was filled on the date after the prescription was received by the pharmacy and taken until the last day supplied. We assumed that extended-release naltrexone was active for 28 days from the date of injection.

Because the MarketScan data do not differentiate between patients who were receiving naltrexone for OUD as opposed to AUD, we further stratified between patients with existing AUD diagnoses at the time of OUD pharmacologic treatment initiation or during the 6 months preceding treatment initiation, and those without recent AUD diagnoses. AUD diagnoses were identified by insurance claims using *ICD-9* and *ICD-10 *codes during any inpatient or outpatient encounter.

For the purposes of sample description, we also obtained data on time-invariant variables, such as age, sex, and insurance status, which were compared among the subsamples with and without recent AUD claims. Because the case-control design allows for individuals to serve as their own control, we did not adjust for these time-invariant variables in our models. However, we included time-varying covariates corresponding to prescriptions for medications commonly found in alcohol and drug poisonings^23^; these included muscle relaxants, antispasmodics, opioids (not buprenorphine or methadone), antidepressants, mood stabilizers, antipsychotics, nonsteroidal anti-inflammatory drugs, and benzodiazepines (eTable 1 in the Supplement). The logic for including these was 2-fold. First, any interaction between these medications and OUD medications may be associated with the point estimates for the OUD medications. Second, use of these medications may serve as proxy variables for comorbidities that can be measured with greater frequency than diagnoses.

### Statistical Analysis

We calculated descriptive statistics for the primary analytical sample using χ^2^ and Kruskal-Wallis tests, which were stratified by recent AUD claims. For our primary analysis, we used a case-control approach described by Allison et al^24^ that includes all observed days limited to 365 days before and after the index alcohol-related acute event already described. Thus, case days included the index event and any subsequent event, whereas control days included all other days within the observation window. An overview of repeated-event, case-control design is illustrated in the eFigure in the Supplement. Models to estimate the odds ratio (OR) describing the association between events and OUD medication exposure days were estimated using conditional logistic regression, with each person serving as the stratification (conditioning) variable. Medication days were coded as 1, and nonmedication days were coded as 0 (reference group). This approach is analogous to a stratified Cox-regression with recurrent events.^24^ As a recurrent-event approach, our analysis allowed for incorporation of time (days since index event) as a covariate, mitigating secular confounding that is often a concern with single-event, case-control studies.^24,25,26^ We controlled for secular time trends using restricted cubic splines.^27^ Unadjusted models included only OUD medications, whereas adjusted models controlled for exposure medications commonly associated with alcohol poisonings.^23^ All *P* values were derived from 2-sided tests, with significance reached at *P* < .05. Statistical analysis was performed using SAS statistical software version 9.4 (SAS Institute) from June 26 through September 28, 2020.

## Results

### Sample and Treatment Characteristics

The final study sample contained 13 335 unique patients with OUD (mean [SD] age, 33.1 [13.1] years; 5884 female participants [44.1%]; mean observation time, 627.6 days; 8 424 214 person-days of observation time) who received OUD medications and had at least 1 alcohol-related acute event. Table 1 summarizes descriptive statistics at the individual participant level, with stratification by the presence of recent AUD claims. In total, 19.6% of the sample (2615 individuals) had claims for more than 1 event. Of all participants, 6299 (47.2%) received buprenorphine in the year before and after the index event; in comparison, 667 (5.0%) received methadone, 1096 (8.2%) received extended-release naltrexone, and 3236 (24.3%) received oral naltrexone. Although all participants had received OUD medication during insurance enrollment, 2037 (15.3%) did not receive OUD medication during the 1 year before and after index event that constituted our study’s observation window.

**Table 1.** Treatment Characteristics at the Individual Participant Level During 1 Year Before and After Index Alcohol-Related Acute Event^a^

Characteristic	Participants, No. (%)			*P* value
	Total (N = 13 335)	OUD with recent AUD claims (n = 7462)	OUD without recent AUD claims (n = 5873)	
OUD medication use during 1 y before and 1 y after index alcohol-related acute event				
Buprenorphine	6299 (47.2)	3134 (42.0)	3165 (53.9)	<.001
Methadone	667 (5.0)	298 (4.0)	369 (6.3)	<.001
Naltrexone extended release	1096 (8.2)	737 (9.9)	359 (6.1)	<.001
Naltrexone oral	3236 (24.3)	2399 (32.2)	837 (14.3)	<.001
None	2037 (15.3)	894 (12.0)	1143 (19.5)	<.001
Use of medications commonly found in association with alcohol poisonings during 1 y before and 1 y after index alcohol-related acute event				
Muscle relaxant	4579 (34.3)	2462 (33.0)	2117 (36.1)	<.001
Antispasmodic	1667 (12.5)	912 (12.2)	755 (12.9)	.27
Opioid (not or buprenorphine or methadone)	8543 (64.1)	4812 (64.5)	3731 (63.5)	.25
Antidepressant	10533 (79.0)	6159 (82.5)	4374 (74.5)	<.001
Mood stabilizer	5891 (44.2)	3790 (50.8)	2101 (35.8)	<.001
Antipsychotic	5938 (44.5)	3521 (47.2)	2417 (41.2)	<.001
Nonsteroidal anti-inflammatory drug	6312 (47.3)	3583 (48.0)	2729 (46.5)	.08
Benzodiazepine	7552 (56.6)	4359 (57.7)	3193 (42.3)	<.001
Sociodemographic characteristics				
Sex				
Male	7451 (55.9)	4235 (56.8)	3216 (54.7)	.02
Female	5884 (44.1)	3227 (43.3)	2657 (45.2)	
Insurance				
Private insurance	9276 (69.6)	5322 (71.3)	3954 (67.3)	<.001
Medicaid	4059 (30.4)	2140 (28.7)	1919 (32.7)	
Year of birth	1977	1975	1979	
Age, mean (SD), y	33.1 (13.1)	34.9 (12.9)	30.8 (13.1)	<.001
Alcohol-related acute events, No.				
1	10 720 (80.4)	5501 (73.7)	5219 (88.9)	<.001
2	1639 (12.3)	1167 (15.6)	472 (8.0)	
≥3	976 (7.3)	794 (10.6)	182 (3.1)	

Abbreviations: AUD, alcohol use disorder; OUD, opioid use disorder.

^a^ This table illustrates OUD treatment characteristics at the individual participant level among those with a history of alcohol-related acute events during the study’s observation window (1 year before and after index event), stratified by recent AUD claims. The prevalence of using specific medications of interest within the study’s observation period is also depicted. The bottom of the table depicts demographic characteristics for participants, as well as number of alcohol-related acute events.

A total of 55.9% of the sample (7462 participants) had a recent AUD claim before or at start of OUD treatment, defined by diagnoses within 6 months of the start of treatment. These patients were less likely to receive agonist therapy than their peers without AUD claims (42.0% [3134 participants] vs 53.9% [3165 participants] for buprenorphine; 4.0% [298 participants] vs 6.3% [369 participants] for methadone). Conversely, they were more likely to receive naltrexone (oral naltrexone, 32.2% [2399 participants] vs 14.3% [837 participants]; extended-release naltrexone, 9.9% [737 participants] vs 6.1% [359 participants]). With regard to sociodemographic characteristics, individuals with recent AUD claims were slightly older (mean [SD] age, 34.9 [12.9] vs 30.8 [13.1] years), more likely to be male (4235 [56.8%] vs 3216 [54.7%] men), less likely to be enrolled in Medicaid (2140 [28.7%] vs 1919 [32.7%] participants), and were more likely to incur multiple alcohol-related events (1967 [26.2%] vs 654 [11.1%] events) (Table 1). Participants with recent AUD claims also had more prescriptions for antidepressants (6156 [82.5%] vs 4374 [74.5%] participants), mood stabilizers (3790 [50.8%] vs 2101 [35.8%] participants), antipsychotics (3521 [47.2%] vs 2417 [41.2%] participants), and benzodiazepines (4359 [57.7%] vs 3193 [42.2%] participants) than participants without AUD claims.

Table 2 depicts the rate of alcohol-related events, per 100 days, stratified by OUD medication exposure and by recent AUD claims. In general, event rates were lower for buprenorphine or methadone treatment days than for days when patients were treated with either naltrexone formulation or for nonmedication days. For nontreatment days, 0.22% of days overall (mean, 535.0 days per person), 0.25% of days for those with active AUD claims (mean, 534.6 days per person), and 0.18% of days among those without active AUD claims (mean, 535.6 days per person) were marked by alcohol-related acute events. For antagonist medications, 0.29% (mean, 15.7 days per person) of oral naltrexone treatment days (0.28% [mean, 8.2 days per person] for those with active AUD claims and 0.29% [mean, 21.5 days per person] for those without active AUD claims) and 0.19% (mean, 6.4 days per person) of extended-release naltrexone treatment days (0.20% [mean, 7.5 days per person] for those with AUD claims and 0.18% [mean, 5.1 days per person] for those without AUD claims) were marked by events. Among agonist medications, 0.11% (mean, 8.4 days per person) of methadone treatment days (0.11% [mean, 5.8 days per person] of days for those with AUD claims and 0.12% [mean, 11.7 days per person] of days for those without AUD claims) and 0.15% (mean, 62.7 days per person) of buprenorphine treatment days (0.13% of days [mean, 48.9 days per person] for those with AUD claims and 0.16% [mean, 80.3 days per person] for those without AUD claims) were marked by alcohol-related acute events.

**Table 2.** Rate of Alcohol-Related Events Associated With OUD Medications, Stratified by Recent AUD Claims^a^

Variable	Total (N = 8 424 214 person-days among 13 335 individuals)		OUD with recent AUD claims (n = 4 629 076 person-days among 7462 individuals)		OUD without recent AUD claims (n = 3 795 138 person-days among 5873 individuals)	
	Mean d/person, No.	Rate of alcohol-related events/100 d	Mean d/person, No.	Rate of alcohol-related events/100 d	Mean d/person, No.	Rate of alcohol-related events/100 d
Treatment						
Buprenorphine	62.7	0.15	48.9	0.13	80.3	0.16
Methadone	8.4	0.11	5.8	0.11	11.7	0.12
Naltrexone extended-release	6.4	0.19	7.5	0.20	5.1	0.18
Naltrexone oral	15.7	0.29	21.5	0.29	8.2	0.28
Nonmedication	535.0	0.22	534.6	0.25	535.6	0.18
All observation days	627.6	0.21	617.5	0.24	640.4	0.18

Abbreviations: AUD, alcohol use disorder; OUD, opioid use disorder.

^a^ This table depicts the percentage of insurance coverage days entailing an alcohol-related acute event, which is compared among days when participants were receiving buprenorphine, methadone, naltrexone (extended release or oral), and nonmedication treatment days. These analyses were further stratified between persons who had recent AUD claims and those without recent AUD claims.

### Association of OUD Medication With Alcohol-Related Acute Events

**Figure 2.**
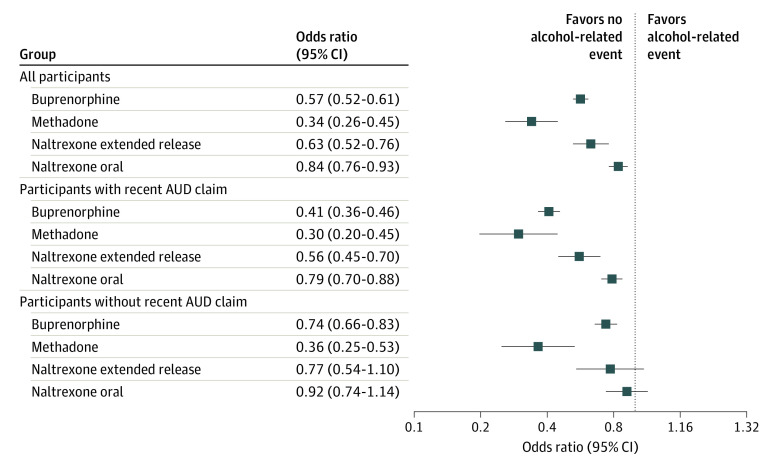
Adjusted Odds of Alcohol-Related Acute Events Associated With Opioid Use Disorder (OUD) Treatment Days Compared With Nontreatment Days Plot shows adjusted odds of alcohol-related acute events associated with OUD treatment days compared with nontreatment days, stratified for all participants, patients with OUD with recent alcohol use disorder (AUD) claims, and patients with OUD without recent AUD claims.

Figure 2 shows the adjusted ORs of admissions for alcohol-related acute events associated with days using OUD medications vs nonmedication days. A full listing of point estimates and 95% CIs is enumerated in eTable 2 (unadjusted models) and eTable 3 (adjusted models) in the Supplement, with the association between OUD medication and alcohol-related acute events unchanged between unadjusted and adjusted analyses. Covariate adjustment had little impact on the other parameters in the model. Among agonist medications, buprenorphine was associated with a 43% reduction (OR, 0.57; 95% CI, 0.52-0.61) and methadone was associated a 66% reduction (OR, 0.34; 95% CI, 0.26-0.45) in adjusted odds of alcohol-related acute events. Among antagonist medication, oral naltrexone was associated with a 37% reduction (OR, 0.63; 95% CI, 0.52-0.76) and extended-release naltrexone was associated with a 16% reduction (OR, 0.84; 95% CI, 0.76-0.93) in the adjusted odds of such events.

In the stratified analysis, buprenorphine’s protective associations against alcohol-related acute events were more pronounced among patients with OUD with recent AUD claims (OR, 0.41; 95% CI, 0.36-0.46) than among peers without such claims (OR, 0.74; 95% CI, 0.66-0.83). In comparison, methadone exhibited similar protective associations in patients with OUD with (OR, 0.30; 95% CI, 0.20-0.45) and without (OR, 0.36; 95% CI, 0.25-0.53) recent AUD claims. The protective associations of extended-release naltrexone (OR, 0.56; 95% CI, 0.45-0.70) and oral naltrexone (OR, 0.79; 95% CI, 0.70-0.88) were significant only among patients with OUD with AUD, as opposed to peers without claims; however, differences in adjusted ORs by AUD status were not significant because of wide 95% CIs among patients with AUD. Across all analyses, the 2 forms of naltrexone did not significantly differ from one another in their association with alcohol-related acute events.

## Discussion

Our study shows that OUD treatment days were associated with reductions in overall odds of admissions for alcohol-related acute events (ie, falls, injuries, and poisonings). We observed risk reductions of 43% for buprenorphine and 66% for methadone treatment days. Extended-release and oral naltrexone treatment days were associated with 37% and 16% reductions, respectively. Naltrexone use was more prevalent among patients with OUD with existing AUD claims than their peers without such claims, consistent with naltrexone’s dual FDA indications of naltrexone for both AUD and OUD treatment. However, naltrexone and buprenorphine showed similar overall protective outcomes against alcohol-related acute events.

Interestingly, our findings illustrate that the greatest overall reduction in alcohol-related acute events were observed with methadone. Time-varying confounding cannot be ruled out because these results can be partly explained by the structure of opioid treatment programs (ie, methadone clinics), which frequently monitor alcohol and illicit drug use with urine drug screens. The effectiveness of OUD medications in AUD may be more limited when it is not accompanied by psychosocial interventions, such as relapse-prevention therapy or counseling focused on medication adherence,^28^ which are seen in opioid treatment programs that dispense methadone. The association between methadone treatment days and alcohol-related events warrants further investigation.

Of note, our results should be interpreted in conjunction with known differences in medication adherence. The true practical effectiveness of antagonist therapies may be hindered by lower medication adherence associated with naltrexone compared with agonist treatments in the OUD population. For instance, substantially greater medication adherence has been observed for buprenorphine treatment (median filled prescriptions of 19 months) compared with both extended-release (9 months) and oral (5 months) naltrexone,^29^ with patients with OUD found to show similar adherence to methadone and buprenorphine.^30^ As our study evaluates ORs that describe protective outcomes associated with medication at a given point in time, the protective outcomes observed in association with buprenorphine and methadone treatment days are likely to be amplified by their higher adherence rates compared with naltrexone. However, our analyses rely on the implicit assumption that risks associated with medication or lack of medication are constant over time. More investigation is needed to evaluate how these risks might vary before, during, and after OUD treatment, in addition to investigating the comparative effectiveness of buprenorphine, methadone, and naltrexone in suppressing alcohol use in patients with OUD.

### Limitations and Strengths

There are several limitations to note. First, although the MarketScan data benefit from a large, national sample with ample longitudinal follow-up, measurement error cannot be ruled out, such that medication coverage does not always reflect actual consumption. Furthermore, patients with OUD without observed AUD claims may have a history of treated or untreated AUD not reflected in recent insurance claims. However, we observed a higher prevalence of naltrexone use in patients with AUD claims, and we also observed significant differences in OUD medication treatment outcomes between patients with OUD with recent AUD claims and peers without such claims, suggesting that the distinction between these 2 groups may be clinically meaningful and thus warrants more research. Second, unmeasured time-varying factors associated with alcohol-related admissions and OUD medication exposure can introduce confounding in case-control designs. We mitigated this, however, by including calendar time and time from event as covariates and restricting participants to 2-year observation periods surrounding the index event in order to reduce heterogeneity in observation time. We also used bidirectional sampling in order to reduce overlap bias resulting from control period selection as a function of event time.^25^ Third, even though self-matching is used by the case-control design, residual confounding by unmeasured time-invariant variables cannot be ruled out. Fourth, the MarketScan data only encompass insured patients with observed alcohol-related acute events, which limits our study’s generalizability.

Despite these limitations, our study was strengthened by its repeated-event, case-control design, allowing for reduction of selection and sampling bias stemming from conventional observational study recruitment. In addition, we controlled for conditions leading to alcohol-related events via adjustment for medications commonly found in association with such events. The protective associations of buprenorphine, methadone, and naltrexone were not significantly changed in the adjusted analyses, providing evidence of robustness to measured confounders.

## Conclusions

The findings of this study suggest that OUD medications are associated with decreased hospital admissions for alcohol-related acute events among persons with OUD. Although naltrexone use was more prevalent among patients with OUD with recent AUD claims, agonist treatments (buprenorphine and methadone) also were associated with fewer alcohol-related events. The present study raises key questions about the potential of OUD medications for reducing alcohol-related events and highlights the need for additional research in this area.

## Notes

Supplement. **eTable 1.** Classification of Medications Commonly Found in Association With Alcohol Poisoning **eTable 2.** Odds of Alcohol-Related Acute Events Associated with OUD Medication Treatment Days Without Adjustment for Treatment Days Encompassing Medications Commonly Found in Association With Alcohol Poisoning **eTable 3.** Odds of Alcohol-Related Acute Events Associated With OUD Medication Treatment Days, With Adjustment for Treatment Days Encompassing Medications Commonly Found in Association with Alcohol Poisoning **eFigure.** Overview of Case-Crossover Design
