# Supplementary material for: Association of Opioid Use Disorder Treatment With Alcohol-Related Acute Events
Source: JAMA Netw Open. 2021 Feb 24;4(2):e210061. doi: 10.1001/jamanetworkopen.2021.0061 (PMC7905500; doi:10.1001/jamanetworkopen.2021.0061)
Supplement: Supplement. — eTable 1. Classification of Medications Commonly Found in Association With Alcohol Poisoning eTable 2. Odds of Alcohol-Related Acute Events Associated with OUD Medication Treatment Days Without Adjustment for Treatment Days Encompassing Medications Commonly Found in Association With Alcohol Poisoning eTable 3. Odds of Alcohol-Related Acute Events Associated With OUD Medication Treatment Days, With Adjustment for Treatment Days Encompassing Medications Commonly Found in Association with Alcohol Poisoning eFigure. Overview of Case-Crossover Design [file jamanetwopen-e210061-s001.pdf]

Supplemental Online Content

Xu KY, Presnall N, Mintz CM, et al. Association of opioid use disorder treatment with alcohol-related acute events. *JAMA Netw Open*. 2021;4(2):e210061. doi:10.1001/jamanetworkopen.2021.0061

eTable 1. Classification of Medications Commonly Found in Association With Alcohol Poisoning

eTable 2. Odds of Alcohol-Related Acute Events Associated with OUD Medication Treatment Days Without Adjustment for Treatment Days Encompassing Medications Commonly Found in Association With Alcohol Poisoning

eTable 3. Odds of Alcohol-Related Acute Events Associated With OUD Medication Treatment Days, With Adjustment for Treatment Days Encompassing Medications Commonly Found in Association with Alcohol Poisoning

eFigure. Overview of Case-Crossover Design

This supplemental material has been provided by the authors to give readers additional information about their work.

© 2021 Xu KY et al. *JAMA Network Open.*

eTable 1. Classification of Medications Commonly Found in Association With Alcohol Poisoning

Opioids		
Fentanyl	Opium	Hydrocodone
Morphine	Propoxyphene	Hydromorphone
Oxycodone	Meperidine	
Oxymorphone	Tramadol	

Muscle Relaxants		
Carisoprodol	Methocarbamol	Metaxalone
Chlorzoxazone	Orphenadrine	Cyclobenzaprine

Nonsteroidal anti-inflammatory drugs (NSAIDs)		
Aspirin	Ketoprofen	Fenoprofen
Diclofenac	Meclofenamate	Naproxen
Diflunisal	Mefenamic	Ibuprofen
Etodolac	Meloxicam	Nabumetone
Oxaprozin	Piroxicam	Sulindac
Tolmetin	Indomethacin	

Antidepressants		
Desipramine	Fluoxetine	Fluvoxamine
Amitriptyline	Bupropion	Mirtazapine
Nortriptyline	Sertraline	Citalopram
Clomipramine	Paroxetine	Escitalopram
Trazodone	Venlafaxine	Duloxetine
Nefazodone	Desvenlafaxine	Vilazodone
Atomoxetine	Phenelzine	Selegiline
Vortioxetine	Tranylcypromine	Levomilnacipran

Mood Stabilizers		
Lithium	Lamotrigine	Carbamazepine
Valproic Acid	Oxcarbazepine	Topiramate

Benzodiazepines		
Alprazolam	Midazolam	Quazepam
Lorazepam	Clonazepam	Zolpidem
Oxazepam	Diazepam	Eszopiclone
Triazolam	Chlordiazepoxide	Zaleplon
Estazolam	Clobazam	
Temazepam	Flurazepam	

© 2021 Xu KY et al. *JAMA Network Open.*

eTable 2. Odds of Alcohol-Related Acute Events Associated With OUD Medication Treatment Days, Without Adjustment for Treatment Days Encompassing Medications Commonly Found in Association With Alcohol Poisoning

		**All Participants (8424214 Person-Days among 13335 Unique Individuals)**		
	Effect	Odds Ratio	95% Confidence Intervals	
Model 2A.1	Buprenorphine Treatment Days	0.56	0.52	0.61
	Methadone Treatment Days	0.34	0.26	0.45
	Naltrexone Extended Release Treatment Days	0.62	0.51	0.74
	Naltrexone Oral Treatment Days	0.79	0.72	0.87
		**OUD with Recent AUD Claims (4629076 Person-Days among 7462 Unique Individuals)**		
	Effect	Odds Ratio	95% Confidence Intervals	
Model 2A.2	Buprenorphine Treatment Days	0.40	0.36	0.45
	Methadone Treatment Days	0.30	0.20	0.45
	Naltrexone Extended Release Treatment Days	0.55	0.44	0.68
	Naltrexone Oral Treatment Days	0.74	0.66	0.83
		**OUD without Recent AUD Claims 3795138 Person-Days among 5873 Unique Individuals)**		
	Effect	Odds Ratio	95% Confidence Intervals	
Model 2A.3	Buprenorphine Treatment Days	0.73	0.65	0.82
	Methadone Treatment Days	0.37	0.25	0.53
	Naltrexone Extended Release Treatment Days	0.75	0.53	1.07
	Naltrexone Oral Treatment Days	0.85	0.68	1.05

eTable 2 shows the unadjusted odds of alcohol-related acute events associated with OUD medication treatment days via three models: model 2A.1 (all participants), model 2A.2 (individuals with OUD and recent AUD claims), and model 2A.3 (individuals with OUD but without recent AUD claims). Odds ratios and 95% confidence intervals are denoted for each variable.

© 2021 Xu KY et al. *JAMA Network Open.*

eTable 3. Odds of Alcohol-Related Acute Events Associated With OUD Medication Treatment Days, With Adjustment for Treatment Days Encompassing Medications Commonly Found in Association with Alcohol Poisoning

		All Participants (8424214 Person-Days among 13335 Unique Individuals)		
	Effect	Odds Ratio	95% Confidence Intervals	
Model 2B.1	Buprenorphine Treatment Days	0.57	0.52	0.61
	Methadone Treatment Days	0.34	0.26	0.45
	Naltrexone Extended Release Treatment Days	0.63	0.52	0.76
	Naltrexone Oral Treatment Days	0.84	0.76	0.93
	Muscle Relaxant Treatment Days	1.15	1.05	1.27
	Antispasmodic Treatment Days	1.08	0.88	1.34
	Opioid (Non Buprenorphine) Treatment Days	0.98	0.92	1.04
	Antidepressant Treatment Days	0.89	0.85	0.93
	Mood stabilizer Treatment Days	0.85	0.79	0.92
	Antipsychotic Treatment Days	0.83	0.78	0.88
	Nonsteroidal anti-inflammatory drug Treatment Days	0.97	0.89	1.05
	Benzodiazepine Treatment Days	1.45	1.37	1.54
		OUD with Recent AUD Claims (4629076 Person-Days among 7462 Unique Individuals)		
	Effect	Odds Ratio	95% Confidence Intervals	
Model 2B.2	Buprenorphine Treatment Days	0.41	0.36	0.46
	Methadone Treatment Days	0.30	0.20	0.45
	Naltrexone Extended Release Treatment Days	0.56	0.45	0.70
	Naltrexone Oral Treatment Days	0.79	0.70	0.88
	Muscle Relaxant Treatment Days	1.17	1.04	1.32
	Antispasmodic Treatment Days	1.04	0.79	1.37
	Opioid (Non Buprenorphine) Treatment Days	1.01	0.93	1.09
	Antidepressant Treatment Days	0.90	0.85	0.95
	Mood stabilizer Treatment Days	0.85	0.77	0.93
	Antipsychotic Treatment Days	0.84	0.78	0.91
	Nonsteroidal anti-inflammatory drug Treatment Days	0.98	0.88	1.08
	Benzodiazepine Treatment Days	1.42	1.32	1.53
		OUD without Recent AUD Claims 3795138 Person-Days among 5873 Unique Individuals)		
	Effect	Odds Ratio	95% Confidence Intervals	
Model 2B.3	Buprenorphine Treatment Days	0.74	0.66	0.83
	Methadone Treatment Days	0.36	0.25	0.53
	Naltrexone Extended Release Treatment Days	0.77	0.54	1.10
	Naltrexone Oral Treatment Days	0.92	0.74	1.14
	Muscle Relaxant Treatment Days	1.10	0.93	1.29

© 2021 Xu KY et al. *JAMA Network Open.*

	Antispasmodic Treatment Days	1.07	0.77	1.50
	Opioid (Non Buprenorphine) Treatment Days	0.93	0.83	1.04
	Antidepressant Treatment Days	0.84	0.78	0.91
	Mood stabilizer Treatment Days	0.84	0.73	0.96
	Antipsychotic Treatment Days	0.78	0.70	0.87
	Nonsteroidal anti-inflammatory drug Treatment Days	0.93	0.80	1.08
	Benzodiazepine Treatment Days	1.44	1.30	1.61

eTable 3 illustrates the adjusted odds of alcohol-related acute events associated with OUD medication treatment days, controlling for treatment days encompassing medications commonly found in association with alcohol poisonings: model 2B.1 (all participants), model 2B.2 (individuals with OUD and recent AUD claims), and model 2B.3 (individuals with OUD but without recent AUD claims).The results in eTable 3 correspond to Table 2 in the text. Odds ratios and 95% confidence intervals are denoted for each variable.

© 2021 Xu KY et al. *JAMA Network Open.*

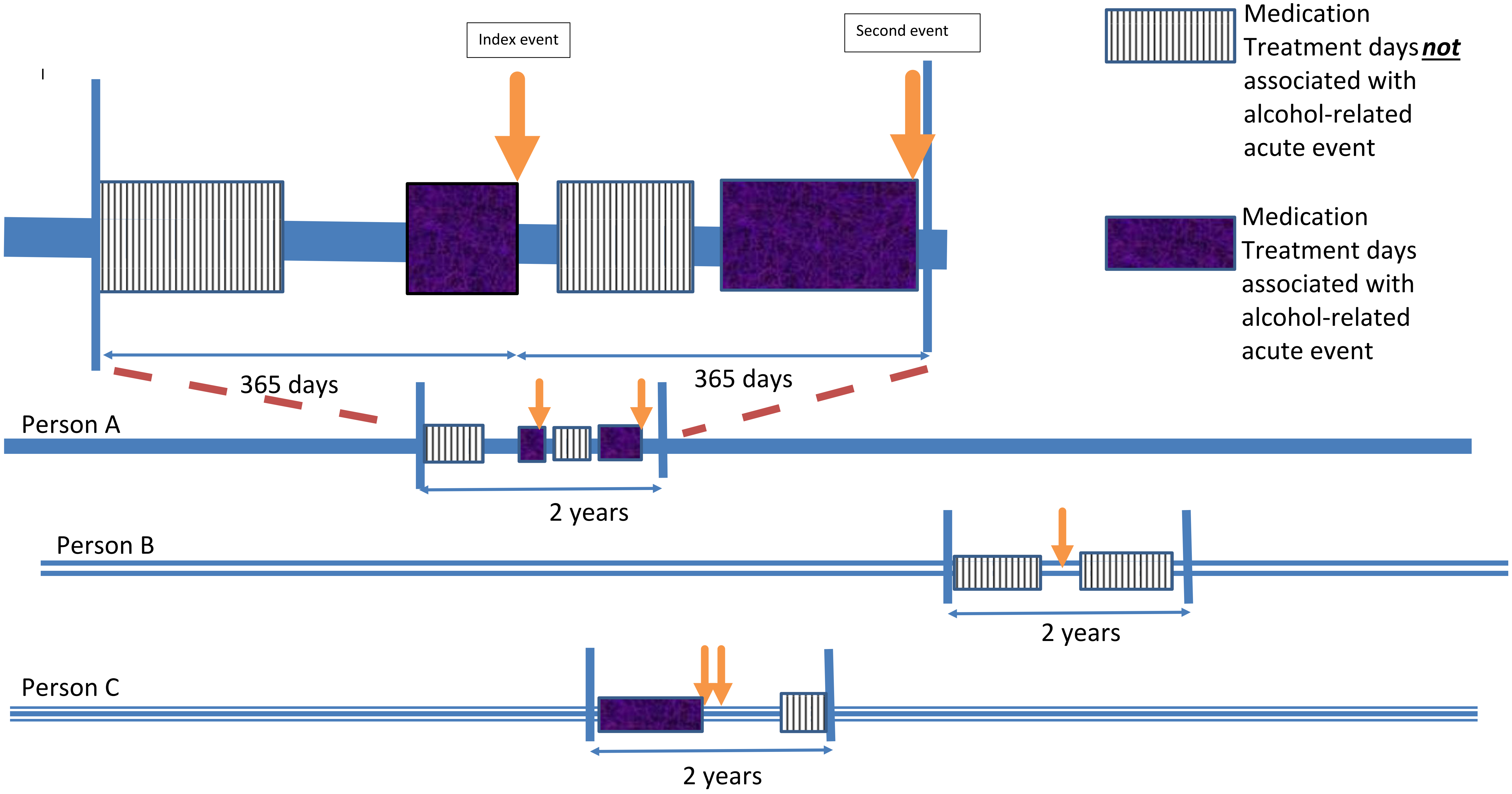

eFigure: Overview of Case-Crossover Design

The eFigure summarizes the study's case-crossover design. Alcohol-related acute events are denoted by arrows. We defined an index event as the first alcohol-related event observed in the data for that individual. An observation period spanning up to a maximum of 1 year before and 1 year after index alcohol-related acute event was selected. Each individual could thus contribute multiple alcohol-related acute events as long as they fell within a maximum of 1 year before and after the index alcohol-related acute event.

© 2021 Xu KY et al. *JAMA Network Open.*

Medication treatment days (encompassing prescriptions such as buprenorphine, methadone, naltrexone ER, or naltrexone oral) are illustrated in the eFigure. Medication treatment days culminating in an alcohol-related acute event are denoted with shaded boxes, whereas treatment days not associated with an alcohol-related acute event are shown by boxes (lines/bars).

For Person A, eFigure 1 shows four medication treatment episodes, two of which culminate in an alcohol-related acute event (index event and a second event), both of which are associated with a medication treatment episode. In comparison, Person B has two medication treatment episodes, neither of which is associated with an alcohol-related acute event. Finally, Person C has two medication treatment episodes, one of which is associated with an alcohol-related acute event (index event); the second alcohol-related acute event is not associated with medication treatment episodes.

© 2021 Xu KY et al. *JAMA Network Open.*
